# Association Between Social Vulnerability and Rates of HIV Diagnoses Among Black Adults, by Selected Characteristics and Region of Residence — United States, 2018

**DOI:** 10.15585/mmwr.mm7105a2

**Published:** 2022-02-04

**Authors:** André F. Dailey, Zanetta Gant, Xiaohong Hu, Shacara Johnson Lyons, Amanda Okello, Anna Satcher Johnson

**Affiliations:** 1 Division of HIV Prevention, National Center for HIV, Viral Hepatitis, STD, and TB Prevention, CDC.

During 2018, Black or African American (Black) persons accounted for 43% of all new diagnoses of HIV infection in the United States (*1*). The annual diagnosis rate (39.2 per 100,000 persons) among Black persons was four times the rate among all other racial/ethnic groups combined, indicating a profound disparity in HIV diagnoses (*1*,*2*). Community-level social and structural factors, such as social vulnerability, might help explain the higher rate of HIV diagnoses among Black persons. Social vulnerability refers to the potential negative health effects on communities caused by external stresses (*3*). CDC used National HIV Surveillance System (NHSS)* and Social Vulnerability Index (SVI)^†^ data to examine the association between diagnosed HIV infections and social vulnerability among Black adults aged ≥18 years. Black adults in communities in the highest quartile of SVI were 1.5 times (rate ratio [RR] = 1.5; 95% CI = 1.4–1.6) as likely to receive a diagnosis of HIV infection as were those in communities in the lowest quartile. Because of a history of racial discrimination and residential segregation, some Black persons in the United States reside in communities with the highest social vulnerability (*4*,*5*), and this finding is associated with experiencing increased risk for HIV infection. The development and prioritization of interventions that address social determinants of health (i.e., the conditions in which persons are born, grow, live, work, and age), are critical to address the higher risk for HIV infection among Black adults living in communities with high levels of social vulnerability. Such interventions might help prevent HIV transmission and reduce disparities among Black adults.

Data on diagnoses of HIV infection among Black adults and reported to CDC through December 2019 were obtained from NHSS. Cases were geocoded to the U.S. Census Bureau tract level based on a person’s residential address at the time of diagnosis. Census tract level social vulnerability data were obtained from the 2018 CDC SVI, which was developed to identify communities with the most potential needs (i.e., highest social vulnerability), before, during, and after public health events. Scores for overall SVI were generated using 15 population-based measures^§^ and were presented as percentile rankings by census tract, with higher scores indicating more vulnerability. SVI scores ranged from 0 to 1 and were categorized as quartiles based on their distribution among all U.S. Census tracts.

NHSS data for Black adults with HIV diagnosed during 2018 were linked with SVI data. Data were analyzed by sex at birth with stratifications by age group and region of residence^¶^ at time of diagnosis to assess differences in HIV diagnosis rates by SVI quartile. HIV diagnosis rates were calculated per 100,000 persons. RRs with 95% CIs were calculated comparing communities with the lowest SVI scores (Quartile 1) to those with the highest scores (Quartile 4) by sex at birth for age group and region of residence. Rates were considered significantly different if the 95% CIs of RRs excluded 1. Differences in numbers of diagnoses across the quartiles were analyzed by sex at birth and transmission category (i.e., male-to-male sexual contact, injection drug use, and heterosexual contact.) Rates and RRs were not calculated for transmission categories because of lack of population data. Data were statistically adjusted using multiple imputation techniques to account for missing HIV transmission categories (*6*). Analyses were conducted using SAS software (version 9.4; SAS Institute, Inc). This activity was reviewed by CDC and was conducted consistent with applicable federal law and CDC policy.**

Among the 13,807 diagnoses of HIV infection among Black adults in 2018, the number and percentage of diagnoses by SVI quartile was 1,045 (7.6%) in Quartile 1; 1,881 (13.6%) in Quartile 2; 3,423 (24.8%) in Quartile 3; and 7,205 (52.2%) in Quartile 4 (Table); SVI scores were missing for 253 persons (1.8%). Black adults in Quartile 4 (rate = 52.1) were 1.5 times (RR = 1.5) as likely to receive a diagnosis of HIV infection compared with those in Quartile 1 (rate = 33.7). In addition, for all within-group comparisons (except for Black persons aged ≥55 years and Black females in the Midwest) there was a higher likelihood of HIV diagnosis in Quartile 4 compared with Quartile 1. Among Black males, the highest disparities in HIV diagnosis rates (i.e., approximately twice as likely in Quartile 4 compared with Quartile 1) were for males aged 45–54 years (RR = 2.3), residing in the Northeast (RR = 2.3) or the West (RR = 2.1). Among males with HIV attributed to male-to-male sexual contact and injection drug use, the number of diagnoses among males in Quartile 4 was 11.6 times the number in Quartile 1. Among Black females, the highest disparities in HIV diagnosis rates (i.e., at least twice as likely in Quartile 4 compared with Quartile 1) were for females aged 18–24 years (RR = 2.0), 35–44 years (RR = 2.4), 45–54 years (RR = 2.0) and those residing in the Northeast (RR = 2.3). Among females with HIV infection attributed to injection drug use, the number of diagnoses in Quartile 4 was 12.3 times the number in Quartile 1.

**TABLE:** Associations between new diagnoses of HIV infection among Black adults and Social Vulnerability Index* of Census tract, by selected characteristics — United States, 2018

Characteristic	Total no. (column %)	Quartile 1 (lowest vulnerability)		Quartile 2		Quartile 3		Quartile 4 (highest vulnerability)		Quartile 4 versus Quartile 1
		No. (row %)	Rate	No. (row %)	Rate	No. (row %)	Rate	No. (row %)	Rate	RR^†^ (95% CI)
**Male (sex at birth)**										
**Age group at diagnosis, yrs**										
18–24	2,950 (28.9)	221 (7.5)	95.8	406 (13.8)	100.4	773 (26.2)	115.4	1,477 (50.1)	145.3	1.5 (1.3–1.7)
25–34	3,985 (39.0)	315 (7.9)	108.2	591 (14.8)	114.2	1,026 (25.7)	120.3	1,998 (50.1)	153.6	1.4 (1.3–1.6)
35–44	1,494 (14.6)	133 (8.9)	44.8	205 (13.7)	45.4	384 (25.7)	54.8	746 (49.9)	72.8	1.6 (1.3–2.0)
45–54	1,010 (9.9)	68 (6.7)	22.5	144 (14.3)	32.5	229 (22.7)	33.7	545 (54.0)	51.8	2.3 (1.8–3.0)
≥55	769 (7.5)	75 (9.8)	18.7	91 (11.8)	14.1	171 (22.2)	16.0	422 (54.9)	22.9	1.2 (1.0–1.6)
**Transmission category^§^**										
Male-to-male sexual contact	8,140 (79.7)	674 (8.3)	—	1,158 (14.2)	—	2,113 (26.0)	—	4,039 (49.6)	—	—
Injection drug use	335 (3.3)	20 (6.1)	—	39 (11.7)	—	78 (23.4)	—	189 (56.4)	—	—
Male-to-male sexual contact and injection drug use	215 (2.1)	10 (4.8)	—	35 (16.1)	—	48 (22.3)	—	116 (53.9)	—	—
Heterosexual contact	1,510 (14.8)	107 (7.1)	—	204 (13.5)	—	341 (22.6)	—	840 (55.6)	—	—
Other	9 (0.1)	0 (3.5)	—	1 (14.0)	—	3 (30.2)	—	5 (52.3)	—	—
**Region of residence**										
Northeast	1,460 (14.3)	75 (5.1)	34.3	169 (11.6)	44.9	324 (22.2)	52.0	876 (60.0)	79.0	2.3 (1.8–2.9)
Midwest	1,539 (15.1)	120 (7.8)	42.6	211 (13.7)	54.4	385 (25.0)	58.0	804 (52.2)	74.2	1.7 (1.4–2.1)
South	6,351 (62.2)	556 (8.8)	64.3	940 (14.8)	66.5	1,659 (26.1)	71.7	3,056 (48.1)	87.4	1.4 (1.2–1.5)
West	858 (8.4)	61 (7.1)	38.8	117 (13.6)	41.5	215 (25.1)	57.6	452 (52.7)	81.8	2.1 (1.6–2.8)
**Subtotal**	**10,208 (100)**	**812 (8)**	**53.3**	**1,437 (14.1)**	**58.4**	**2,583 (25.3)**	**65.0**	**5,188 (50.8)**	**83.1**	**1.6 (1.4–1.7)**
**Female (sex at birth)**										
**Age group at diagnosis, yrs**										
18–24	478 (13.3)	27 (5.6)	13.4	59 (12.3)	15.8	101 (21.1)	15.9	284 (59.4)	27.1	2.0 (1.4–3.0)
25–34	946 (26.3)	65 (6.9)	23.2	100 (10.6)	19.6	204 (21.6)	24.1	557 (58.9)	37.0	1.6 (1.2–2.1)
35–44	866 (24.1)	48 (5.5)	15.6	112 (12.9)	22.8	227 (26.2)	30.0	465 (53.7)	37.6	2.4 (1.8–3.3)
45–54	689 (19.1)	48 (7.0)	15.2	88 (12.8)	18.1	160 (23.2)	20.8	381 (55.3)	30.4	2.0 (1.5–2.7)
≥55	620 (17.2)	45 (7.3)	9.6	85 (13.7)	10.4	148 (23.9)	10.5	330 (53.2)	13.0	1.4 (1.0–1.8)
**Transmission category^§^**										
Injection drug use	264 (7.3)	13 (4.9)	—	32 (12.2)	—	56 (21.0)	—	160 (60.8)	—	—
Heterosexual contact	3,315 (92.1)	218 (6.6)	—	409 (12.3)	—	780 (23.5)	—	1,847 (55.7)	—	—
Other	20 (0.6)	2 (10.9)	—	3 (13.9)	—	5 (24.4)	—	10 (49.3)	—	—
**Region of residence**										
Northeast	630 (17.5)	25 (4.0)	11.9	86 (13.7)	21.3	133 (21.1)	18.9	383 (60.8)	27.0	2.3 (1.5–3.4)
Midwest	440 (12.2)	44 (10.0)	16.2	41 (9.3)	10.0	103 (23.4)	14.4	243 (55.2)	18.0	1.1 (0.8–1.5)
South	2,251 (62.5)	147 (6.5)	15.4	278 (12.4)	17.3	530 (23.5)	20.0	1,248 (55.4)	29.6	1.9 (1.6–2.3)
West	278 (7.7)	17 (6.1)	12.5	39 (14.0)	15.2	74 (26.6)	21.1	143 (51.4)	23.8	1.9 (1.1–3.1)
**Subtotal**	**3,599 (100)**	**233 (6.5)**	**14.8**	**444 (12.3)**	**16.6**	**840 (23.3)**	**19.0**	**2,017 (56.0)**	**26.6**	**1.8 (1.6–2.1)**
**Total^¶^**	**13,807 (100)**	**1,045 (7.6)**	**33.7**	**1,881 (13.6)**	**36.6**	**3,423 (24.8)**	**40.8**	**7,205 (52.2)**	**52.1**	**1.5 (1.4–1.6)**

**Abbreviations:** RR = rate ratio; SVI = Social Vulnerability Index.

* SVI scores represent percentile rankings by Census tract, ranging from 0–1, with higher scores indicating more vulnerability. Scores were categorized into quartiles based on distribution among all U.S. Census tracts. https://www.atsdr.cdc.gov/placeandhealth/svi/index.html

^†^ Two rates are statistically different if the 95% CI does not include 1.0.

^§^ Numbers have been adjusted for missing transmission category and rounded to integers. Rates and RRs for transmission categories were not calculated because of lack of population data.

^¶^ Total includes 253 cases without SVI rankings.

## Discussion

During 2018, the rate of new HIV diagnoses per 100,000 population among Black adults was higher in communities with the highest SVI (Quartile 4; 52.1) than in communities with the lowest SVI (Quartile 1; 33.7). Approximately one half (52.2%) of Black adults with newly diagnosed HIV infection resided in the most socially vulnerable census tracts, which are often racially segregated communities comprising predominately Black persons (*5*,*7*). The social and economic marginalization of Black persons, including residential segregation, is correlated with factors associated with higher social vulnerability and higher rates of HIV diagnosis (*7*). Residential segregation contributes to higher rates of HIV diagnosis and poor health outcomes among Black persons because isolation limits access to important resources and affects neighborhood quality; populations residing in lower-income and relatively more isolated areas experience vulnerability to negative health outcomes, including HIV infection (*5*,*7*,*8*). In addition, persons lacking basic economic and social support in communities with higher social vulnerability are more likely to be overwhelmed by routine life demands (e.g., addressing issues with unstable housing or unable to take time off from minimum-wage job because of lack of paid leave (*9*). Although social vulnerability does not explain all the disparity in HIV diagnosis (*5*), Black adults in communities with the highest social vulnerability might find it harder to obtain HIV prevention and care services because of various factors, such as poverty, limited access to health care, substance use disorder, transportation to services, housing insecurity, HIV stigma, racism, discrimination, and high rates of sexually transmitted diseases (*7*,*10*). These factors directly and indirectly affect the health of Black adults with HIV infection and those who experience risk for infection (*10*).

The findings in this report are subject to at least four limitations. First, data on diagnoses of HIV infection might not be representative of all persons with HIV because not all persons with HIV have been tested or tested at a time when the infection could be detected and diagnosed. Second, because results of anonymous and self-tests are not reported, surveillance case reports might not include all persons who received positive HIV test results. Third, testing patterns are influenced by many factors, including the extent to which testing is routinely offered to specific groups and the availability of, and access to, medical care and testing services. Finally, HIV infection might have occurred in a place other than the person’s residence at the time of diagnosis.

HIV strategies, interventions, and programs that address the needs and challenges of Black adults in communities with the highest social vulnerability are needed. The development and prioritization of interventions that address social determinants of health^††^ (i.e., the conditions in which persons are born, grow, live, work, and age), are critical to addressing the higher risk for HIV infection among Black adults living in communities with high levels of social vulnerability. Such interventions might help prevent HIV transmission and reduce disparities among Black adults.

**Summary**

**What is already known about this topic?**

In 2018, Black persons accounted for nearly one half of all new diagnoses of HIV infection in the United States. The annual diagnosis rate among Black persons was four times the rate among all other racial or ethnic groups combined.

**What is added by this report?**

Rates of new HIV diagnoses among Black adults were higher in communities with the highest social vulnerability. Approximately one half of Black adults with diagnosed HIV reside in the upper quartile of socially vulnerable U.S. Census tracts in the United States.

**What are the implications for public health practice?**

Intensified prevention efforts are needed to reduce HIV transmission among Black persons in communities with the highest social vulnerability.

## Footnotes

* NHSS is the primary source for monitoring HIV trends in the United States. Assisted by CDC, state and local health departments collect and report deidentified data regarding HIV infection cases to CDC.

†
https://www.atsdr.cdc.gov/placeandhealth/svi/index.html

§ The 15 population-based social factors incorporated into SVI measures were from four domains: 1) socioeconomic status (based on poverty, employment, income, and educational attainment); 2) household composition and disability (based on age [pediatric and elderly populations], civilians aged >5 years with a disability, and single-parent households); 3) racial and ethnic minority residents (i.e., do not identify as non-Hispanic or Latino White) and English proficiency (based on representation of racial and ethnic minority residents and actual proficiency); and 4) housing type and transportation (based on multiunit structures, mobile homes, crowding, no household vehicle access, and institutionalized group quarters). https://www.atsdr.cdc.gov/placeandhealth/svi/index.html

¶ U.S. Census Bureau regions: *Northeast:* Connecticut, Maine, Massachusetts, New Hampshire, New Jersey, New York, Pennsylvania, Rhode Island, and Vermont; *Midwest:* Illinois, Indiana, Iowa, Kansas, Michigan, Minnesota, Missouri, Nebraska, North Dakota, Ohio, South Dakota, and Wisconsin; *South*: Alabama, Arkansas, Delaware, District of Columbia, Florida, Georgia, Kentucky, Louisiana, Maryland, Mississippi, North Carolina, Oklahoma, South Carolina, Tennessee, Texas, Virginia, and West Virginia; *West:* Alaska, Arizona, California, Colorado, Hawaii, Idaho, Montana, Nevada, New Mexico, Oregon, Utah, Washington, and Wyoming.

** 45 C.F.R. part 46.102(l)(2), 21 C.F.R. part 56; 42 U.S.C. Sect. 241(d); 5 U.S.C. Sect. 552a; 44 U.S.C. Sect. 3501 et seq.

††
https://www.cdc.gov/socialdeterminants/docs/sdh-white-paper-2010.pdf
